# Predicting variation in word decoding development in deaf and hard-of-hearing children

**DOI:** 10.1007/s11145-023-10444-0

**Published:** 2023-05-12

**Authors:** Sascha Couvee, Loes Wauters, Harry Knoors, Ludo Verhoeven, Eliane Segers

**Affiliations:** 1 Behavioural Science Institute, Radboud University, Thomas van Aquinostraat 4, Nijmegen, 6525 GD The Netherlands; 2 Royal Kentalis, Van Vollenhovenlaan, Utrecht, 659-661, 3527 JP The Netherlands

**Keywords:** Deaf and hard-of-hearing children, Word decoding development, Phonological awareness, Letter knowledge, Rapid automatized naming, Verbal short-term memory

## Abstract

Background: Deaf and hard-of-hearing (DHH) children may experience difficulties in word decoding development. Aims: We aimed to compare and predict the incremental word decoding development in first grade in Dutch DHH and hearing children, as a function of kindergarten reading precursors. Methods and procedures: In this study, 25 DHH, and 41 hearing children participated. Kindergarten measures were phonological awareness (PA), letter knowledge (LK), rapid naming (RAN), and verbal short-term memory (VSTM). Word decoding (WD) was assessed at three consecutive time points (WD1, 2, 3) during reading instruction in first grade. Outcomes and results: The hearing children scored higher than the DHH children on PA and VSTM only, although the distribution of WD scores differed between the groups. At WD1, PA and RAN predicted WD efficiency in both groups; but PA was a stronger predictor for hearing children. At WD2, LK, RAN, and the autoregressor were predictors for both groups. While at WD3, only the autoregressor was a significant predictor. Conclusions and implications: WD development in DHH children on average shows similar levels as in hearing children, though within the DHH group more variation was observed. WD development in DHH children is not as much driven by PA; they may use other skills to compensate.

Learning to read is fundamental for academic success. Deaf and hard-of-hearing (DHH) children may experience difficulties in learning to read. A recent study showed that while DHH children in kindergarten scored similar to a norm-group of typically hearing children on English word decoding, they were lagging behind after a few months in first grade (Antia et al., 2020). How word decoding in DHH children develops during the very first few months of reading instruction compared to typically hearing children, and how this can be predicted from kindergarten measures has not yet been studied. In typically hearing children, key precursor measures for word decoding development are phonological awareness, letter knowledge, and rapid automatized naming (RAN) (e.g., Caravolas et al., 2012; Landerl et al., 2022; Moll et al., 2014; Verhoeven & Perfetti, 2022). Furthermore, verbal short-term memory (verbal STM) is an important cognitive ability related to reading (Melby-Lervåg et al., 2012; Swanson et al., 2009). In the current study, we compared word decoding development in DHH and typically hearing children as a function of these precursors during the first six months of first grade.

## Word decoding development

Learning to read involves systematic acquisition of mappings between phonemes and graphemes (Castles et al., 2018). The triangle model of reading, a connectionist model, provides a framework for (skilled) word reading (Harm & Seidenberg, 2004; Plaut et al., 1996). This triangular model includes connections between the orthography (the graphemes), the phonology (the phonemes) and semantics. The early stages of reading are characterized by forming connections between orthography and phonology, while skilled word reading relies more on connections between orthography and semantics. When going from decoding to more fluent reading, semantic retrieval becomes important. RAN, a measure of lexical retrieval, is thought to measure some aspect of the connection between orthography and phonology in the triangle model, as it also requires an arbitrary association between visual and phonological stimulus, similar to grapheme-phoneme correspondences (Manis et al., 1999). In line with this framework, the meta-analysis by Melby-Lervåg et al. (2012) indeed showed that, phonological skills (phonological awareness, verbal STM) and grapheme (letter) knowledge predict reading skills (Melby-Lervåg et al., 2012; see also Landerl et al., 2022).

One of the most important predictors for word decoding skills in typically hearing children is phonological awareness (Melby-Lervåg et al., 2012). Phonological awareness refers to the ability to attend to, reflect upon, and manipulate the different sounds of a word (e.g., Goswami, 2001). Letter knowledge refers to the knowledge of grapheme-phoneme correspondences, and together with phonological awareness, forms the basis of learning to read in alphabetic writing systems (Byrne, 1998). RAN refers to the ability to sequentially name lists of objects, digits, letters, or colours as quickly as possible and is a measure for lexical retrieval. RAN has been shown to predict unique variance in reading above and beyond phonological awareness (e.g., Manis et al., 1999) and has been mostly linked to reading fluency (Landerl et al., 2019; Moll et al., 2014; Verhoeven & Perfetti, 2022). A large study involving different orthographies showed that verbal STM, a measure of phonological processing ability, contributed to both word reading speed and accuracy (Moll et al., 2014).

When children learn to read in a language with a transparent orthography, word decoding accuracy scores quickly are at ceiling (Landerl & Wimmer, 2008; Schaars et al., 2017). Word decoding speed, however, increases during word decoding development (Landerl & Wimmer, 2008; Verhoeven & van Leeuwe, 2009), even as word decoding tasks become more difficult with the inclusion of more graphemes during the first months of reading instruction (Schaars et al., 2017). In the current study, we investigated word decoding efficiency, a combination of accuracy and speed. Only few studies examined reading development in typically hearing children during the first months of reading instruction. These studies showed that word reading accuracy and speed develop from the beginning of reading instruction, although speed develops mainly after a basic level of accuracy is reached, and that word decoding skills can be predicted from kindergarten phonological awareness, letter knowledge, RAN (Juul et al., 2014; Schaars et al., 2017), as well as verbal STM (Schaars et al., 2017).

## Word decoding development in deaf and hard-of-hearing children

Written language is based on spoken language. Due to their limited access to spoken language, DHH children are at risk for developing reading problems (Perfetti & Sandak, 2000). Reasoning from the triangle model, the phonology node is impaired for children who are DHH. Previous studies with DHH children or DHH adults yielded mixed findings concerning the activation of phonology during reading (Blythe et al., 2018; Mayberry et al., 2011; Ormel et al., 2010). However, many studies have shown that the development of reading skills is delayed in DHH children compared to typically hearing children (e.g., Antia et al., 2020). Evidence from two longitudinal studies on reading development of beginning DHH readers in the United Kingdom showed that their word decoding skills already lagged behind when children were in kindergarten or first grade (Harris et al., 2017; Kyle & Harris, 2011). In both studies, the delay in word decoding skills in DHH children increased with every assessment. In a study conducted in the United States, the reading progress of beginning DHH readers was measured twice a year (autumn and spring), in kindergarten and first grade, and accuracy scores were compared to established norms for typically hearing children (Antia et al., 2020). In kindergarten, most of the DHH children scored within these norms on word decoding accuracy, while in the autumn of first grade, about one-third of the DHH children scored below the norm for typically hearing children. From the beginning of reading instruction, word decoding thus seems a difficult task for DHH children learning to read in English, with its highly opaque orthography.

However, a study involving older DHH children in mainstream schools in Ireland, aged seven to 13, showed that the majority of the DHH children had word decoding scores within the normal range, although there was a lot of variation (Mathews & Donnell, 2020). Furthermore, a study on reading, phonological and orthographic abilities in Spanish, which has a transparent orthography, showed that deaf children who received a cochlear implant (CI) before 2;6 were not delayed in word reading compared to typically hearing children (Domínguez et al., 2019).

## Precursor measures for reading in deaf and hard-of-hearing children

The known precursors of word decoding development in typically hearing children, have also been studied in DHH children. Spoken phonological awareness is more difficult for DHH children, as their ability to hear speech sounds is limited, the development of phonological awareness skills is, therefore, impaired (Johnson & Goswami, 2010), also in case of early cochlear implantation (Nittrouer et al., 2014). DHH children scored lower on phonological awareness tasks compared to norms for typically hearing children, in both kindergarten and first grade (Antia et al., 2020). The delay in phonological awareness between DHH and typically hearing children increased from kindergarten to first grade, but it was not studied whether phonological awareness predicted reading development. In other studies, phonological awareness has been shown to correlate with concurrent word decoding (Johnson & Goswami, 2010; Kyle & Harris, 2011) in 5;6–7;6-year-old DHH children and word decoding two years later (Harris et al., 2017). However, phonological awareness did not predict a significant amount of variance over and above age, vocabulary, and speechreading (Harris et al., 2017). In a cross-sectional study, Domínguez et al. (2019) showed that phonological skills in Spanish DHH children between six and 18 years old largely contributed to their performance on a reading task, similar to typically hearing children.

The second precursor, letter knowledge, is often age-appropriate in DHH children (e.g., Ambrose et al., 2012; Easterbrooks et al., 2008). Studies with beginning DHH readers show that letter knowledge was related to concurrent word decoding (Cupples et al., 2014) and predicted word decoding measured two years later (Kyle & Harris, 2011). In a study by Herman and colleagues (2019), although involving older children, letter knowledge was associated with nonword decoding in 11-year-old DHH children.

The third precursor, RAN, has received less attention in relation to word decoding development in DHH children. Nittrouer and colleagues (2012) investigated emergent literacy in deaf children with CIs and typically hearing children aged six-seven. The DHH children with CIs performed similarly on RAN as typically hearing children, but RAN did not predict word decoding accuracy in either of the groups. In two recent studies in older DHH children, aged 10–18, RAN explained a unique amount of variance in word reading accuracy (Herman et al., 2019; Sun et al., 2022).

Finally, regarding verbal STM, according to Harris et al. (2013), verbal STM in deaf children with CIs is delayed compared to age-matched typically hearing peers. However, another study found no difference between deaf and typically hearing children on verbal STM, but the deaf children were older than the typically hearing children (Kyle & Harris, 2011). Verbal STM neither correlated with concurrent word decoding nor predicted word decoding one or two years later in either group. Other studies found deaf children with CIs or hearing aids (HAs) scored lower on a verbal STM task compared to typically hearing peers (Johnson & Goswami, 2010; Nittrouer et al., 2012), and verbal STM did not predict word reading accuracy. However, verbal STM did correlate moderately to strongly to word decoding in 9-11-year-old DHH children (Herman et al., 2019; Johnson & Goswami, 2010). Evidence on verbal STM and its relation to word decoding in DHH children is thus mixed.

To summarize, previous studies on word decoding in DHH children have mostly been conducted with children learning to read English, a highly opaque orthography. Moreover, only few studies have looked at the prediction of reading precursors on word decoding development in DHH children. Furthermore, most studies focused on groups of children with a large age range, possibly obscuring effects at play in beginning DHH readers. Additionally, previous studies investigated word decoding accuracy or speed, but not efficiency, which combines both accuracy and speed. Finally, none of the previous studies involving DHH children have measured word decoding development during reading instruction, from the first graphemes to the point where all graphemes have been learned. When starting to follow the reading development from the very beginning, early identification of reading problems is possible.

## Current study

The current study aimed to compare the incremental development of word decoding in DHH and typically hearing children while learning to read in Dutch, a transparent orthography, during the first six months of first grade, as a function of kindergarten precursor measures phonological awareness, letter knowledge, RAN, and verbal STM. We aimed to study to what extent these key precursors for word decoding development in typically hearing children are also relevant in DHH children. To investigate word decoding development and its relation to the precursors, we posed the following research questions:

1. In what ways do DHH and typically hearing children differ in kindergarten precursor measures and incremental word decoding development in first grade?
2. To what extent can the incremental development of word decoding in first grade be predicted from the precursor measures phonological awareness, letter knowledge, RAN, and verbal STM in kindergarten, and does this prediction differ for DHH and typically hearing children?

Regarding the first research question, we hypothesized that DHH children and typically hearing children differ in their phonological awareness and verbal STM, but based on previous research, not in their letter knowledge and RAN. As most studies found delays in word decoding already in first grade, we expected differences on word decoding from the beginning, with the DHH children scoring lower than the typically hearing children. Regarding the second research question it was hypothesized that the same precursors would predict word decoding in both groups, although the strength of the prediction might differ between the groups. This was investigated exploratory.

## Method

The current study was approved by the Ethics Committee of the Social Sciences at the Radboud University [ECSW-2018-179].

### Participants

#### Deaf and hard-of-hearing children

In this study, 25 DHH children (16 boys, 9 girls; *M*_age_ 6;3 (*SD* 6.58 months)) and 41 typically hearing children (24 boys, 17 girls; *M*_age_ 5;9 (*SD* 3.84 months)) participated (see Table 1 for the characteristics of each DHH child). Participants were recruited through schools and itinerant services. All parents gave active consent. The study started when all children were at the end of their second year of kindergarten, which is a two-year programme in the Netherlands. The study continued until all participants were halfway first grade. The group of DHH children was older than the group of typically hearing children, *t*(64)=-4.37, *p* < .001, *d* = 0.07. Although differing in age, all children received formal reading instruction from the start of the first grade, which was the same point in time for all children. The DHH children were from special schools (*n* = 21) and mainstream schools (*n* = 4). Five DHH children were from multilingual backgrounds, other than Sign Supported Dutch (SSD) or Sign Language of the Netherlands (SLN).

**Table 1:** Characteristics of the DHH participants

#ID	Age at testing	Gender	Hearing loss	Hearing devices	Length of use hearing device	Home language	Level of education reporting parent
1	5;07	Female	Missing	HA	5;6	Dutch, SSD, SLN	Upper secondary
2	5;07	Female	Severe-profound	HA	5;6	Dutch	Upper secondary
3	5;08	Female	Profound	CI	Missing	Dutch, SSD, SLN	College/University
4	5;09	Male	Profound	CI	4;9	Dutch	College/University
5	5;09	Female	Profound	CI	1;0	Dutch, SSD	College/University
6	5;10	Male	Profound	CI	5;9	Dutch, SSD	College/University
7	5;11	Male	Severe	HA	5;7	Dutch, SSD	Upper secondary
8	6;01	Male	Mild-profound	HA	1;0	Dutch, SSD	College/University
9	6;01	Male	Profound	CI	4;6	Dutch	Upper secondary
10	6;01	Male	Profound	CI	4;8	Dutch	Lower secondary
11	6;02	Female	Profound	CI	4;4	SSD	College/University
12	6;03	Male	Profound	CI	3;7	Dutch, SSD	Lower secondary
13	6;04	Female	Severe	HA	Missing	Dutch	Lower secondary
14	6;04	Female	Profound	CI	Missing	Dutch, SSD	Upper secondary
15	6;05	Male	Severe-profound	HA	Missing	Dutch, German, SSD	College/University
16	6;06	Male	Profound	CI	Missing	Dutch, SSD	Upper secondary
17	6;07	Male	Profound	CI	5;7	Dutch	College/University
18	6;08	Male	Mild-moderately severe	HA	5;9	Dutch, Turkish	Lower secondary
19	6;08	Male	Severe-moderate	BAHA	Missing	Dutch	College/University
20	6;08	Male	Profound	CI	6,1	Dutch, SSD	College/University
21	6;09	Female	Profound	CI	5;1	English, Telugu	College/University
22	6;10	Male	Moderately severe	HA	5;8	Dutch	Lower secondary
23	6;10	Male	Profound	CI	5;8	Dutch, SSD	Lower secondary
24	6;11	Female	Profound	CI	3;8	Pashto, SSD	Upper secondary
25	8;01	Male	Profound	CI	0;10	Kurdish, SSD	Unknown

*Note.* CI = Cochlear Implant; HA = Hearing Aid; BAHA = Bone Anchored Hearing Aid;

SSD = Sign Supported Dutch; SLN = Sign Language of the Netherlands.

The typically hearing children were recruited from three mainstream schools across the Netherlands. The parents of the typically hearing children reported their highest finished level of education: 7.3% of the parents received lower secondary education, 22.0% received upper secondary education, and 70.8% were educated at college/university. The groups did not differ on the level of education of the reporting parent, *U* = 361.5, *p* = .06. All typically hearing children had Dutch as home language, two children also spoke Russian or Turkish in addition to Dutch.

### Instruments

#### Precursor measures

The precursor measures for reading were administered at the end of kindergarten.

### Phonological awareness

#### Phoneme isolation

We measured phoneme isolation skills with a task in which the first phoneme of a high frequency CVC-structured, monosyllabic word had to be repeated (Schaars et al., 2017). All items were presented orally. The task comprised 10 items. The reliability of the task was good (Cronbach’s *a* = 0.89, calculated on the current sample).

#### Phoneme segmentation

Phoneme segmentation skills were measured with a task in which a high frequent, monosyllabic word had to be segmented in phonemes (Schaars et al., 2017). The task was administered orally and comprised 10 items which increased in difficulty; the first items were CVC-structured words while the last items were CCVC- or CVCC-structured words. The reliability of the task was good (Cronbach’s *a* = 0.89, calculated on the current sample).

#### Rhyme

Passive rhyme was measured with the rhyme task of the *Screeningsinstrument Beginnende Geletterdheid* (Diagnostic Instrument for Emerging Literacy; Vloedgraven et al., 2009). The task comprised two practice items and 15 test items. The task was presented on a laptop, each item consisted of three pictures presented on the laptop screen, while an audio file was played asking: “What rhymes with…”. The child had to point to the picture that rhymed with the word in the audio file. The reliability of the task was acceptable (Cronbach’s *a* = 0.77, calculated on the current sample).

A principal component analysis showed the phonological awareness tasks to have high loadings on one component: phoneme isolation (0.83), phoneme segmentation (0.86), and rhyme (0.84). The rhyme task was scaled to 10, as the task comprised of more items than phoneme isolation and phoneme segmentation. The phonological awareness score was the sum of all phonological awareness tasks.

#### Letter knowledge

Letter knowledge was measured through a passive grapheme-phoneme correspondence task. The task comprised 22 graphemes, excluding c, q, x, y and the digraphs. Children were asked: “Where is the /m/ of milk?”. The child had to point to one of four graphemes presented on paper. The graphemes were printed in lower case in four squares on a card. Arial (Monotype, Microsoft) font type of size 180 was used because it is similar to the font used in the reading curricula. The reliability of the task was good (Cronbach’s *a* = 0.87, calculated on the current sample).

#### Rapid automatized naming

We measured RAN with a lexical retrieval task of objects (Schaars et al., 2017). The children were asked to name the pictures as quickly and accurately as possible during one minute. DHH children were also allowed to use signs. The task comprised five pictures of objects, all corresponding with monosyllabic high frequent words in Dutch (saw, pot, thumb, trousers, and tent). The pictured objects were repeated over six columns of 22 pictures. We calculated split-half reliability on the even and uneven test items. The reliability of the task was excellent (Spearman-Brown Coefficient = 0.99, calculated on the current sample).

#### Verbal short-term memory

To test verbal STM we used a task from the *Testinstrumentarium Taalontwikkelingsstoornissen* (“Test Instruments for Developmental Language Disorders”, Verhoeven et al., 2013). In this task, children were asked to repeat strings of words, in the same order. The strings of words increased in length, from two to seven words, and was presented on a laptop on which the audio files were played. The task comprised one practice item, consisting of three words, and 12 test items. All words included in the task were high frequent monosyllabic words. The task was terminated if the child answered two items of the same length incorrectly. The reliability was acceptable (Cronbach’s *a* = 0.75, calculated on the current sample). As this task did not test pronunciation, responses of the DHH children that resembled the targets were also accepted as correct.

### Systematic reading instruction in first grade

All participating schools used a systematic reading instruction method starting in first grade, giving daily reading instruction lessons. Reading instruction started directly after the summer holiday. Among the schools, three different reading methods were used; *Veilig Leren Lezen* (“Learning to Read Safely”; Mommers et al., 2003) was used at seven schools: four special schools for DHH children (*n* = 14) and three mainstream schools for typically hearing children (*n* = 41). *Lijn 3* (“Line 3”; Malmberg, 2014) was used at six schools with DHH children (*n* = 8): two schools for special education (*n =* 4) and four mainstream schools (*n* = 4). *Lees en Beslis* (“Read and Decide”; Quadvlieg, 2002) was used at one special school for DHH children (*n* = 3).

All three reading methods provide systematic reading instruction, which teach all 34 graphemes required for reading in Dutch, during the first half of first grade. Veilig Leren Lezen and Lijn 3 use an incremental, systematic, phonics-based approach, and Lees en Beslis uses an incremental, systematic, orthography-based approach. In all three reading methods, children are gradually taught all grapheme-phoneme correspondences, in short monosyllabic words.

#### Curriculum embedded word decoding tasks

Children who received Veilig Leren Lezen reading instruction were tested with curriculum embedded word decoding tasks. In these tasks, word decoding was assessed with 40 monosyllabic CV/CVC/VC-structured words, which had to be read aloud as accurately and quickly as possible during one minute. The word decoding tasks included all taught grapheme-phoneme correspondences, meaning that with every task the set of grapheme-phoneme correspondences increased. These word decoding tasks are part of the curriculum of Veilig Leren Lezen, but are not used in Lijn 3 and Lees en Beslis. To test all children in a similar manner, three similar curriculum embedded word decoding tasks were for Lijn 3 and Lees en Beslis. The occurrences of the graphemes in every word decoding task was counterbalanced. Across reading methods, all word decoding tasks included more or less the same amount of graphemes (see Table 2).

**Table 2:** Overview of grapheme-phoneme correspondences tested in the word decoding tasks

⇓ Time after beginning of school year		WD1	VLL, hearing *n* = 41, DHH *n* = 11	i k m s p aa r e v n t ee b oo
	6–8 weeks		Lijn 3, DHH *n* = 8	r s k e aa n i d t m oo ee b ie a
			L & B, DHH *n* = 3	r k n s d p m t o i e a
		WD2	VLL, hearing *n* = 41, DHH *n* = 13	i k m s p aa r e v n t ee b oo d oe z ij h w o a u j
	12–16 weeks		Lijn 3, DHH *n* = 8	r s k e aa n i d t m oo ee b ie a au ei eu f g h l o oe p u w
			L & B, DHH *n* = 3	r k n s d p m t o i e a aa b g h l oo u v z
	18–24 weeks	WD3	VLL, hearing *n* = 38, DHH *n* = 14	i k m s p aa r e v n t ee b oo d oe z ij h w o a u j eu ie l ou uu g au ui f ei
			Lijn 3, DHH *n* = 8	r s k e aa n i d t m oo ee b ie a au ei eu f g h l o oe p u w ij j ou ui uu v z

Incremental grapheme-phoneme exposure◊

*Note.* WD = word decoding task, VLL = Veilig Leren Lezen, L & B = Lees en Beslis.

All word decoding tasks consisted of high frequency words and words that were part of the reading method. All words were CV/CVC/VC-structured. Words for the tasks for Lijn 3 and Lees en Beslis were chosen from the curriculum or from the *Streeflijst Woordenschat voor Zesjarigen* (Target List Vocabulary for Six-year-olds; Schaerlaekens et al., 2000). The latter is a word list with teacher ratings of the percentage of children in kindergarten and first grade expected to know a certain word. We used words from this list that were expected to be known by at least 75% of the children in first grade. To test whether the words in the word decoding tasks for Veilig Leren Lezen, Lijn 3, and Lees en Beslis were comparable, we conducted Kruskal-Wallis Tests to compare the ratings from the Streeflijst Woordenschat voor Zesjarigen (Schaerlaekens et al., 2000) for the words. The three word decoding tasks (WD1, 2, and 3) did not differ on the percentage of kindergartners and first-graders assumed to know the words, for WD1: *H*(2) = 3.15, *p* = .21, for WD2: *H*(2) = 3.21, *p* = .20, and for WD3: *U* = 554, *p* = .16.

### Procedure

In kindergarten, the children were tested towards the end of the school year with three individual assessments, which took place at two different days. These assessments were part of a larger study, in which other tasks were also administered, not included in this study. The precursor measures were administered by the first author of this paper and four trained research assistants. The tasks were administered in the following order: letter knowledge, phonological awareness, and RAN on the first day, verbal STM on the second day. Instructions were provided in Dutch for typically hearing children and DHH children in mainstream schools, since the latter ones did not use SSD or SLN at school. For the DHH children in special schools, instructions were given in SSD, as they were used to receiving instructions in SSD. The instructions were repeated if necessary. All tasks were administered in spoken language, responses were required in spoken language, except for rhyme and letter knowledge, which required pointing, and RAN, for which participants were also allowed to sign (though only two DHH children responded with signs). Tasks were administered individually in a separate room. Some DHH children used frequency modulation (FM) systems (*n* = 9) during the individual assessments, to play the speech of the experimenter or the sound of the laptop directly on their CIs or HAs. The volume of the laptop was set at 70 dB prior to testing for all children. For the DHH children, adjustments were made to the volume if required, with a maximum volume of 75 dB.

The first grade word decoding tasks were administered by the (remedial) teacher at school, after instructions by the first author. The tasks were administered individually. For the school that used Lees en Beslis, it was not possible to administer WD3 as the school was closed due to the COVID-19 pandemic. See Table 2 for an overview of when the children were tested.

### Analyses

For the analyses we used IBM SPSS (version 27). To answer the first research question, group differences were tested with Mann-Whitney U Tests, as scores on phonological awareness, letter knowledge, verbal STM and WD1 and 3 for the typically hearing children were not normally distributed. For the DHH children, scores on letter knowledge, verbal STM and WD1 were not normally distributed. Group differences on RAN and WD2 were tested with independent samples t-tests. To test whether the groups increased their word decoding scores, a two-way mixed ANOVA was conducted, with group as a between-subjects factor and time as within-subjects factor. We report the results with the Huynh-Feldt correction, as Mauchly’s test of sphericity indicated that sphericity had been violated, χ^2^(2) = 17.92, *p* < .001. Post hoc tests are reported with Bonferroni correction. We conducted correlation analyses to investigate the relations between the tasks per participant group. We report Pearson correlations for the normally distributed tasks and Spearman correlations for the other tasks (see Table 5).

For the second research question, on the prediction of word decoding, we conducted linear regression analyses, with phonological awareness, letter knowledge, RAN, and verbal STM as independent variables and the word decoding tasks as the dependent variables. For WD2 and 3 we included the previous word decoding task as autoregressor. In step 1 of the regression analyses we entered group and the tasks, while in step 2 we entered the interaction terms of group and the tasks. If step 2 was not a significant improvement of the model, we only report step 1 of the regression analysis.

## Results

### Group differences

To answer the first research question, we compared the groups on the precursors and word decoding. The groups differed on phonological awareness, *U* = 151.5, *p* = < 0.001, *η*^*2*^ = 0.35, and verbal STM, *U* = 58, *p* = < 0.001, *η*^*2*^ = 0.57, with the typically hearing children scoring higher on both tasks. No differences were found on letter knowledge, *U* = 408, *p* = .17, *η*^*2*^ > 0.01, RAN, *t*(64) = 1.06, *p* = .29, *g*_*Hedge*_=0.27, WD1, *U* = 385, *p* = .34, *η*^*2*^ = 0.01, WD2, *t*(63)=-1.14, *p* = .26, *g*_*Hedges*_=-0.29, and WD3, *U* = 416.5, *p* = .98, *η*^*2*^ > 0.01 (see Table 3).

**Table 3:** Descriptive statistics for DHH children and hearing children

Task	DHH children					Hearing children					
	*n*	*m*	*SD*	*Mdn*		*n*	*m*	*SD*	*Mdn*	*Max. score*	*p*
Phonological awareness	25	11.41	5.56	12		41	19.74	5.68	21.33	30	< .001
Letter knowledge	25	17.76	4.73	20		41	16.85	4.13	18	22	.16
RAN	25	35.40	11.46	37		41	37.98	8.27	38	132	.29
Verbal STM	25	1.28	1.34	1		41	4.41	1.41	4	12	< .001
WD1	22	15.41	12.18	12		41	15.29	7.94	14	40	.34
WD2	24	23.75	11.83	23		41	20.85	8.51	21	40	.26
WD3	22	24.64	13.16	24		38	24.29	10.06	21	40	.98

*Note*. Means, standard deviations and medians are based on raw scores.

Regarding word decoding, descriptive results suggest that the distribution of the scores between the groups differs. Some DHH children scored at the top end at all WD tasks, while other DHH children scored very low. Variation exists in the typically hearing group as well, however, it was more normally distributed, while for WD1 and WD2 the distribution for the DHH group was more dichotomous. This was also evidenced by Levene’s test, which indicated unequal variances between the groups for all three tasks; WD1, *F*(1,54) = 8.70, *p* = .005, WD2, *F*(1,54) = 8.28, *p* = .004, WD3, *F*(1,54) = 5.26, *p* = .026. Also, standard deviations for the DHH group were larger than for the typically hearing group, thus evidencing more variation within the DHH children. There was no significant interaction between group and word decoding task, *F*(1.62, 87.55) = 0.07, *p* = .90, *η*_*p*_^*2*^ = 0.001. There was a main effect of time; the word decoding scores increased with every measurement for the whole group *F*(1.62, 87.55) = 39.39, *p* < .001, see Table 4 for differences between the word decoding tasks. There was no significant effect of group, *F*(1.54) = 0.72, *p* = .40.

**Table 4:** Repeated measures ANOVA for word decoding for the total sample

	Difference in mean	*p*		95% CI					
WD 1–2	-6.12	< .001		[-8.53,-3.71]					
WD 1–3	-9.28	< .001		[-12.52,-6.04]					
WD 2–3	-3.16	.001		[-5.25,-1.07]					

With regard to the second research question, the correlation analysis (Table 5) revealed several significant correlations. For the DHH children, WD1 did not have any significant correlations with the precursor measures. WD2 and WD3 correlated strongly with letter knowledge and RAN. Furthermore, all word decoding tasks were strongly intercorrelated.

**Table 5:** Correlations for DHH and hearing children

		Phonological awareness	*p*	Letter knowledge	*p*	RAN	*p*	Verbal STM	*p*	WD1	*p*	WD2	*p*	WD3	*P*
1.	Phonological awareness	-		.62	< .001	.40	.051	.54	.005	.18	.416	.37	.077	.42	.052
2.	Letter knowledge	.59	< .001	-		.24	.240	.48	.015	.50	.019	.61	.001	.57	.006
3.	RAN	.35	.025	.12	.442	-		.28	.171	.23	.294	.50	.013	.71	< .001
4.	Verbal STM	.25	.119	.02	.910	.20	.216	-		− .05	.840	.63	.002	.18	.425
5.	WD1	.55	< .001	.33	.034	.39	.012	.20	.208	-		.77	< .001	.75	.001
6.	WD2	.51	.001	.43	.005	.39	.012	.24	.136	.76	< .001	-		.91	< .001
7.	WD3	.50	.001	.45	.005	.32	.049	.07	.684	.50	< .001	.79	< .001	-	

*Note.* Correlations for DHH children above the diagonal, correlations for hearing children below the diagonal. Pearson correlations are reported for the DHH children on phonological awareness and RAN/WD2/WD3, and RAN and WD2/WD3, and for the hearing children on RAN and WD2. For all other correlations we report Spearman’s rho correlation.

For the typically hearing children, all precursor measures had moderate to strong correlations to the word decoding tasks, except for verbal STM, which did not have any significant correlation with the word decoding tasks. Finally, all word decoding tasks were intercorrelated.

### Predicting word decoding development

Regarding the second research question, the analyses showed no differences between the groups in which precursor measures predicted word decoding. Only for WD1, phonological awareness was a stronger predictor for typically hearing children, as there was an interaction between phonological awareness and group (Table 6). However, in both groups, phonological awareness and RAN predicted WD1, explaining 65% of the variance. For WD2, letter knowledge and RAN predicted a significant amount of variance in both groups, even when the autoregressive effect of WD1 was high (Table 7), together explaining 64% of the variance. There were no significant interactions between group and the tasks; step 2 of the regression analysis was not a significant improvement of the model for WD2. For WD3, none of the precursors explained a significant amount of variance over and above the autoregressor in both groups (Table 8). The model explained 72% of the variance in WD3. There was no significant interaction between group and the tasks in step 2 of the regression analysis.

**Table 6:** Predictors for word decoding task 1 for the total sample (*n*=63)

	Independent variable	*B*	*SE B*	β	*p*	95% CI	*ΔR* ^*2*^
Step 1	Group	3.23	3.83	.16	.40	[-4.47,10. 29]	.28^**^
	Phonological awareness	.26	.25	.18	.30	[-.24,.75]	
	Letter knowledge	.68	.30	.31	.03	[.08,1.28]	
	RAN	.24	.14	.23	.08	[-.03,.51]	
	Verbal STM	.26	.89	.05	.77	[-1.52,2.04]	
Step 2	Group	6.26	7.84	.31	.43	[-9.50,22.03]	.42^***^
	Phonological awareness	.44	.17	.31	.01	[.10,.77]	
	Letter knowledge	.40	.22	.18	.08	[-.05,.84]	
	RAN	.29	.11	.27	.02	[.06,.52]	
	Verbal STM	.64	.67	.14	.35	[-.71,1.98]	
	Phonological awareness x group	1.04	.19	1.07	< .001	[.65,1.42]	
	Letter knowledge x group	− .12	.23	− .18	.61	[-.59,.35]	
	RAN x group	− .29	.25	− .55	.27	[-.80,.23]	
	Verbal STM x group	-2.19	1.39	− .21	.12	[-4.98,.60]	

*Note: R*^2^ final model = .71, *R*^2^_adj_ final model = .65, *f*^2^ final model = 2.45

^*^p < .05; ^**^p < .01, ^***^p < .001

**Table 7:** Predictors for word decoding task 2 for the total sample (*n*=65)

Independent variable	*B*	*SE B*	β	*p*	95% CI	*ΔR* ^*2*^
Group	2.22	3.00	.10	.46	[-3.81,8.26]	.68^***^
Phonological awareness	− .01	.18	− .01	.94	[-.38,.35]	
Letter knowledge	.52	.23	.22	.03	[.06,.98]	
RAN	.24	.11	.20	.03	[.03,.45]	
Verbal STM	.27	.67	.05	.69	[-1.08,1.62]	
WD1	.65	.10	.59.	< .001	[.44, 86]	

*Note: R*^2^ final model = .68, *R*^2^_adj_ final model = .64, *f*^2^ final model = 2.13

^***^p < .001

**Table 8:** Predictors for word decoding task 3 for the total sample (*n* = 60)

Independent variable	*B*	*SE B*	β	*p*	95% CI	*ΔR* ^*2*^
Group	− .58	3.04	-,02	.85	[-6.69,5.53]	.74^***^
Phonological awareness	.17	.18	.10	.35	[-.20,.54]	
Letter knowledge	.12	.25	.04	.65	[-.39,.62]	
RAN	.20	.12	.14	.10	[-.04,.44]	
Verbal STM	− .67	.68	− .12	.33	[-2.03,.70]	
WD2	.83	.11	.75	< .001	[.61,1.05]	

*Note: R*^2^ final model = .78, *R*^2^_adj_ final model = .72, *f*^2^ final model = 3.55

^***^p < .001

## Discussion

Our aim was to compare and predict the incremental word decoding development in DHH and typically hearing children learning to read in Dutch, and predict their word decoding development (WD1, 2, 3) from kindergarten reading precursors. Concerning the first research question, the results mostly support the hypotheses. As expected, typically hearing children scored higher on the auditory tasks, phonological awareness and verbal STM, but there were no differences between DHH and typically hearing children on letter knowledge and RAN. This is in line with previous research (e.g., Ambrose et al., 2012; Antia et al., 2020; Easterbrooks et al., 2008; Harris et al., 2013; Johnson & Goswami, 2010; Nittrouer et al., 2012).

We expected differences on word decoding between the groups. However, on average, the DHH and typically hearing children did not differ on word decoding. It is noteworthy though, that in our sample, for WD1, 27% of the DHH children scored below 1 SD below the mean for typically hearing children. For WD2, this was 21%, and for WD3 it was 32%. While we did not find differences at group level, a considerable part of the DHH children scored below the means of the typically hearing children. In the study by Antia et al. (2020), also about one-third of the DHH children scored 1 SD below norms for typically hearing children. Interestingly, also a considerable amount of DHH children scored more than 1 SD above the means for typically hearing children; for WD1 this was 23%, for WD 2 it was 38%, and for WD3 it was 36%. Furthermore, we investigated word decoding efficiency, while Antia et al. (2020) looked at word decoding accuracy. Research shows that for children learning to read in Dutch accuracy scores are generally high from the start, while word decoding efficiency increases until Grade 6 (Schaars et al., 2017; Verhoeven et al., 2022). Our study shows that efficiency already increases during the first half of Grade 1. Furthermore, our results show that some DHH children acquire word decoding skills very quickly and accurately, while other DHH children still struggle after six months of formal reading instruction. Possible explanations for the differences within the DHH group could be related to differences in reading instruction, child characteristics or school setting (mainstream or special education), however future studies should investigate this further.

As for the second research question, regarding the prediction of word decoding development, we expected that the same precursors would predict word decoding in both groups. In line with the triangle model, the beginning stages of learning to read are characterized by making connections between orthography and phonology (Harm & Seidenberg, 2004; Plaut et al., 1996). Indeed, we found the same precursors to predict word decoding, namely phonological awareness, letter knowledge and RAN, although the strength of the prediction of phonological awareness differed between the groups. This is noteworthy as the DHH children might not be able to establish connections between orthography and phonology in the same way as typically hearing children. However, DHH children might use sign language phonology as a form of compensation (Brysbaert, 2022; Lederberg et al., 2019; Segers & Verhoeven, 2015). Verbal STM did not predict word decoding in either of the groups.

Regarding phonological awareness, we found differences between DHH and typically hearing children on the prediction of the first word decoding task; phonological awareness predicted word decoding in both groups, but phonological awareness was a stronger predictor for the typically hearing group. Phonological awareness predicted word decoding in the DHH participants, who had all used hearing amplification, most from a very young age, and were exposed to spoken language. Furthermore, there was a strong association between phonological awareness and letter knowledge in the DHH children. This differs from Harris et al. (2017); phonological awareness did not predict word decoding one or two years later in their sample of DHH children, however, concurrent phonological awareness at the third measurement did predict word decoding in their study.

Letter knowledge predicted WD2 in both DHH and typically hearing children. Furthermore, the correlation between letter knowledge and WD1 within the DHH group was marginally significant (*p* = .052), indicating a possible relation between kindergarten letter knowledge and word decoding already from the first measurement. Letter knowledge in kindergarten could be an important precursor for later word decoding development. Already many special schools for DHH children start practicing some letter knowledge skills in kindergarten, to get a bit of a head start in Grade 1.

RAN predicted word decoding in both groups at the first two measurements (WD1 and WD2). It thus seems that RAN is as important for learning to read for DHH children as it is for typically hearing children, although when looking at the correlations, RAN seems to pick up a bit later for the DHH group. The relevance of RAN is noteworthy, as it involves the fast retrieval of a phonological form from memory, and phonological skills are often less well-developed or precise in DHH children (e.g., Nittrouer et al., 2014). It is important to note that the DHH children were allowed to respond in sign language, but only two children did. Apparently, despite their hearing loss, DHH children are not hampered in their lexical retrieval skills and these skills also relate to their word decoding efficiency, and not only accuracy which was found in previous studies (Herman et al., 2019; Nittrouer et al., 2012; Sun et al., 2022).

We did not find evidence of a relation between verbal STM and word decoding in DHH children. This finding is in line with previous studies (Kyle & Harris, 2011; Nittrouer et al., 2012). For typically hearing children, verbal STM and WD1 correlated. The verbal STM task was difficult for the DHH children, as evidenced by their low mean score. This restriction of range may explain why we did not find a relation between verbal STM and word decoding. Some studies did find a relation between verbal STM and word decoding (Herman et al., 2019; Johnson & Goswami, 2010), but the participants in these studies were older.

Our study is one of the few studies to investigate early word decoding development in beginning DHH readers. On average, we found no differences between DHH and typically hearing children on word decoding in first grade, though some DHH children struggled with word decoding skills, while others had above-average word decoding skills. Additionally, we found evidence for different mechanisms to learning to read; the effect of kindergarten precursors differed between the groups. Phonological awareness was a stronger predictor for word decoding development in the typically hearing children. Letter knowledge and RAN were important predictors of word decoding for both DHH, and typically hearing children. Finally, previous word decoding tasks predicted scores on the subsequent word decoding task, showing high stability in word decoding skills in both groups. Indicating that also for DHH children, word decoding development can be predicted from the beginning of formal reading instruction, similar as has been shown for typically hearing children (e.g., Schaars et al., 2017). In our study, we did not find that as a group, DHH children were delayed in their word decoding development compared to typically hearing children.

Despite the positive finding that there were no differences between group averages on word decoding, the hearing loss of the DHH children is likely to impact their reading development. Our study shows that, in line with the triangle model of reading, both orthography (as measured by letter knowledge), phonology (as measured by phonological awareness), and RAN are important for early word decoding development in DHH and typically hearing children (Harm & Seidenberg, 2004; Manis et al., 1999; Plaut et al., 1996). However, as children who are DHH do not have full access to phonology, their connections between orthography and phonology likely differ from typically hearing children. The phonology part of the triangle is constrained, which might lead to other connections between the nodes in DHH children and a different reading development (see also Segers & Verhoeven, 2015). This constraint on phonology is also evidenced by our finding of a weaker prediction of phonological awareness to WD1 in DHH children. Furthermore, in the beginning stages of learning to read it is especially important to establish strong connections between orthography and phonology, in order to become a skilled reader and then make stronger connections between orthography and semantics. This likely does not only influence word reading fluency, but also later reading comprehension, as for good reading comprehension, word reading has to be efficient and effortless, with flexible representations of the orthography, phonology and semantics of a word, as stated by the Lexical Quality Hypothesis (Perfetti & Hart, 2002). At least some DHH children may use a different route to learning to read (e.g., Lederberg et al., 2019). This is corroborated by a recent study on the Lexical Quality Hypothesis with deaf and typically hearing adults, which showed that reading comprehension in deaf adults was predicted only by orthographic and semantic knowledge, while for the typically hearing adults phonological awareness was a strong predictor (Sehyr & Emmorey, 2022). Furthermore, DHH children who use sign language may use sign language phonology as a compensatory mechanism in their reading development (Keck & Wolgemuth, 2020; Lederberg et al., 2019; Segers & Verhoeven, 2015).

### Limitations and future directions

A limitation of the current study is the sample of the DHH group; most children were enrolled in special education; four children were in mainstream education. Furthermore, due to the Covid-19 pandemic, it was not possible to recruit a larger sample of DHH children. A larger sample size would have allowed for more advanced statistical models and more comparisons within the DHH group. Future studies should seek to include more DHH children, as well as a more balanced sample of DHH children.

The current study investigated the predictive relation of known precursor measures for reading in typically hearing children also in DHH children. It may very well be the case that DHH children use compensatory mechanisms to develop word decoding, such as speechreading or fingerspelling, (Harris et al., 2017; Lederberg et al., 2019). Especially since the mean word decoding scores did not differ between the groups despite the difference in phonological awareness, we expect some compensatory skills in the DHH group. However, that was not the focus of the current study, in which we made the direct comparison with typically hearing children. Future studies should investigate the effect of such variables on word decoding in DHH children. Furthermore, future studies should investigate the further development of word decoding skills, as we have only investigated the very beginning. Studies show that differences between DHH and typically hearing children in word decoding increase with age (Antia et al., 2020; Kyle & Harris, 2011). An interesting future direction would also be to study the reciprocal relation of learning to read and phonological awareness in DHH children, as they likely develop their phonological awareness skills during reading instruction. Also, despite the positive outcome that word decoding skills, on average, do not differ between DHH and typically hearing children, the relation between word decoding and reading comprehension should be investigated, as previous studies with DHH children have shown a gap between the two; where the level of word decoding skills was higher than the level of reading comprehension (Mathews & Donnell, 2020; Wauters et al., 2006).

### Implications

Our study provides a number of implications for practice. Firstly, letter knowledge, phonological awareness, and RAN are important skills for later word decoding, and they develop already prior to formal reading instruction. Children with weaker precursors may be at risk for delays in word decoding development. Secondly, despite the positive outcome that on average DHH children and typically hearing children do not differ on their word decoding abilities during the first six months of reading instruction, a larger part of DHH compared to typically hearing children have not yet mastered word decoding skills after acquiring all graphemes. These children should receive extra support to develop word decoding skills.

## Conclusions

To conclude, DHH and typically hearing children only differ on auditory precursors, and word decoding development does not differ between the groups at the beginning of first grade, even with the majority of the DHH children enrolled in special education. However, more variation in word decoding scores was observed within the DHH group, more DHH children struggle with word decoding development compared to typically hearing children, while other DHH children perform above average. Our study shows similar precursors for word decoding in DHH and typically hearing children, but also provides evidence for different mechanisms in learning to read in the two groups, as phonological awareness was a stronger predictor in the typically hearing group.

## References

[CR1] Ambrose SE, Fey ME, Eisenberg LS (2012). Phonological awareness and print knowledge of preschool children with cochlear implants. Journal of Speech Language and Hearing Research.

[CR2] Antia SD, Lederberg AR, Easterbrooks S, Schick B, Branum-Martin L, Connor CM, Webb MY (2020). Language and reading progress of young deaf and hard-of-hearing children. Journal of Deaf Studies and Deaf Education.

[CR3] Blythe HI, Dickins JH, Kennedy CR, Liversedge SP (2018). Phonological processing during silent reading in teenagers who are deaf/hard of hearing: An eye movement investigation. Developmental Science.

[CR4] Brysbaert, M. (2022). Word recognition II: Orthography-phonology. In M. J. Snowling, C. Hulme, & K. Nation (Eds.), *The Science of Reading: A Handbook* (Second Edi, pp. 79–101). Wiley. 10.1002/9781119705116.ch4

[CR5] Byrne, B. (1998). *The foundations of literacy*. Psychology Press.

[CR6] Caravolas M, Lervåg A, Mousikou P, Efrim C, Litavský M, Onochie-Quintanilla E, Salas N, Schöffelová M, Defior S, Mikulajová M, Seidlová-Málková G, Hulme C (2012). Common patterns of prediction of literacy development in different alphabetic orthographies. Psychological Science.

[CR7] Castles A, Rastle K, Nation K (2018). Ending the reading wars: Reading acquisition from novice to expert. Psychological Science in the Public Interest.

[CR8] Cupples L, Ching TYC, Crowe K, Day J, Seeto M (2014). Predictors of early reading skill in 5-year-old children with hearing loss who use spoken language. Reading Research Quarterly.

[CR9] Domínguez A, Alegría J, Carrillo M, González V (2019). Learning to read for spanish-speaking deaf children with and without cochlear implants: The role of phonological and orthographic representation. American Annals of the Deaf.

[CR10] Easterbrooks SR, Lederberg AR, Miller EM, Bergeron JP, McDonald Connor C (2008). Emergent literacy skills during early childhood in children with hearing loss: Strengths and weaknesses. Volta Review.

[CR12] Goswami, U. (2001). Early phonological development and the acquisition of literacy. In S. B. Neuman, & D. K. Dickinson (Eds.), *Handbook of early Literay Research* (pp. 111–125). The Guilford Press.

[CR13] Harm MW, Seidenberg MS (2004). Computing the meanings of words in reading: Cooperative division of labor between visual and phonological processes. Psychological Review.

[CR14] Harris MS, Kronenberger WG, Gao S, Hoen HM, Miyamoto RT, Pisoni DB (2013). Verbal short-term memory development and spoken language outcomes in deaf children with cochlear implants. Ear and Hearing.

[CR15] Harris M, Terlektsi E, Kyle FE (2017). Concurrent and longitudinal predictors of reading for deaf and hearing children in primary school. Journal of Deaf Studies and Deaf Education.

[CR16] Herman R, Kyle FE, Roy P (2019). Literacy and phonological skills in oral deaf children and hearing children with a history of dyslexia. Reading Research Quarterly.

[CR17] Johnson C, Goswami U (2010). Phonological awareness, vocabulary, and reading in deaf children with cochlear implants. Hearing Research.

[CR18] Juul H, Poulsen M, Elbro C (2014). Separating speed from accuracy in beginning reading development. Journal of Educational Psychology.

[CR19] Keck, T., & Wolgemuth, K. (2020). American Sign Language phonological awareness and English reading abilities: Continuing to explore new relationships. *Sign Language Studies*, *20*(2), 334–354. 10.1353/sls.2020.0004 For

[CR20] Kyle FE, Harris M (2011). Longitudinal patterns of emerging literacy in beginning deaf and hearing readers. Journal of Deaf Studies and Deaf Education.

[CR23] Landerl K, Wimmer H (2008). Development of word reading fluency and spelling in a consistent orthography: An 8-year follow-up. Journal of Educational Psychology.

[CR22] Landerl K, Freudenthaler HH, Heene M, De Jong PF, Desrochers A, Manolitsis G, Parrila R, Georgiou GK (2019). Phonological awareness and rapid automatized naming as longitudinal predictors of reading in five alphabetic orthographies with varying degrees of consistency. Scientific Studies of Reading.

[CR21] Landerl K, Castles A, Parrila R (2022). Cognitive precursors of reading: A cross-linguistic perspective. Scientific Studies of Reading.

[CR24] Lederberg AR, Branum-Martin L, Webb MY, Schick B, Antia S, Easterbrooks SR, Connor CMD (2019). Modality and interrelations among language, reading, spoken phonological awareness, and fingerspelling. Journal of Deaf Studies and Deaf Education.

[CR25] Malmberg (2014). *Lijn 3* https://www.malmberg.nl/basisonderwijs/methodes/lezen/lijn-3.htm

[CR26] Manis FR, Seidenberg MS, Doi LM (1999). See Dick RAN: Rapid naming and the Longitudinal Prediction of Reading Subskills in First and Second Graders. Scientific Studies of Reading.

[CR27] Mathews ES, Donnell MO (2020). Phonological decoding and reading comprehension in deaf and hard-of-hearing children. European Journal of Special Needs Education.

[CR28] Mayberry RI, del Giudice AA, Lieberman AM (2011). Reading achievement in relation to phonological coding and awareness in deaf readers: A meta-analysis. Journal of Deaf Studies and Deaf Education.

[CR29] Melby-Lervåg M, Lyster SAH, Hulme C (2012). Phonological skills and their role in learning to read: A meta-analytic review. Psychological Bulletin.

[CR30] Moll K, Ramus F, Bartling J, Bruder J, Kunze S, Neuhoff N, Streiftau S, Lyytinen H, Leppänen PHT, Lohvansuu K, Tóth D, Honbolygó F, Csépe V, Bogliotti C, Iannuzzi S, Démonet JF, Longeras E, Valdois S, George F, Landerl K (2014). Cognitive mechanisms underlying reading and spelling development in five european orthographies. Learning and Instruction.

[CR31] Mommers, C. L., Verhoeven, L., Koekebacker, E., van der Linden, S., Stegeman, W., & Warnaar, J. (2003). Veilig leren lezen. Zwijsen.

[CR32] Nittrouer S, Caldwell A, Lowenstein JH, Tarr E, Holloman C (2012). Emergent literacy in kindergartners with cochlear implants. Ear & Hearing.

[CR33] Nittrouer S, Sansom E, Low K, Rice C, Caldwell-Tarr A (2014). Language structures used by kindergartners with cochlear implants: Relationship to phonological awareness, lexical knowledge and hearing loss. Ear & Hearing.

[CR34] Ormel E, Hermans D, Knoors H, Hendriks A, Verhoeven L (2010). Phonological activation during visual word recognition in deaf and hearing children. Journal of Speech Language and Hearing Research.

[CR35] Perfetti, C. A., & Hart, L. (2002). The lexical quality hypothesis. In L. Verhoeven, C. Elbro, & P. Reitsma (Eds.), *Precursors of functional literacy* (11 vol., pp. 189–213). John Benjamins.

[CR36] Perfetti CA, Sandak R (2000). Reading optimally builds on spoken language: Implications for deaf readers. Journal of Deaf Studies and Deaf Education.

[CR37] Plaut DC, McClelland JL, Seidenberg MS, Patterson K (1996). Understanding normal and impaired word reading: Computational principles in quasi-regular domains. Psychological Review.

[CR38] Quadvlieg, T. (2002). *Lees en beslis: Methode voor aanvankelijk lezen van dove en slechthorende kinderen*. Koninklijke Ammanstichting, Rudolf Mees Instituut.

[CR39] Schaars MMH, Segers E, Verhoeven L (2017). Word decoding development in incremental phonics instruction in a transparent orthography. Reading and Writing.

[CR40] Schaerlaekens, A. M., Kohnstamm, G. A., & Lejaegere, M. (2000). *Streeflijst woordenschat zesjarigen*. Pearson Benelux.

[CR41] Segers, E., & Verhoeven, L. (2015). Benefits of technology-enhanced learning for deaf and hard-of-hearing students. In *Educating Deaf Learners* (pp. 481–502). 10.1093/acprof:oso/9780190215194.003.0021

[CR42] Sehyr ZS, Emmorey K (2022). Contribution of Lexical Quality and sign Language variables to reading comprehension. Journal of Deaf Studies and Deaf Education.

[CR43] Sun, P., Zhao, Y., Chen, H., & Wu, X. (2022). Contribution of linguistic skills to word reading in DHH students. *Journal of Deaf Studies and Deaf Education*, *enac003*, 1–14. 10.1093/deafed/enac003 10.1093/deafed/enac003 35405012

[CR44] Swanson HL, Zheng X, Jerman O (2009). Working memory and reading disabilities: A selective meta-analysis of the literature. Journal of Learning Disabilities.

[CR45] Verhoeven L, Perfetti C (2022). Universals in learning to read across languages and writing systems. Scientific Studies of Reading.

[CR47] Verhoeven L, van Leeuwe J (2009). Modeling the growth of word-decoding skills: Evidence from dutch. Scientific Studies of Reading.

[CR46] Verhoeven, L., Keuning, J., Horsels, L., & van Boxtel, H. (2013). *Verantwoording Testinstrumentarium Taalontwikkelingsstoornissen (T-TOS)*. Cito.

[CR48] Verhoeven L, Voeten M, Keuning J (2022). Modeling developmental changes in print tuning in a transparent alphabetic orthography. Frontiers in Neuroscience.

[CR49] Vloedgraven, J., Keuning, J., & Verhoeven, L. (2009). *LOVS Screeningsinstrument Beginnende Geletterdheid Groep 2 en 3* Handleiding. Cito.

[CR50] Wauters LN, Van Bon WHJ, Tellings AEJM (2006). Reading comprehension of dutch deaf children. Reading and Writing.

